# Long-term survival of young women receiving fertility-sparing surgery for ovarian cancer in comparison with those undergoing radical surgery

**DOI:** 10.1038/bjc.2011.394

**Published:** 2011-10-04

**Authors:** H Kajiyama, K Shibata, M Mizuno, T Umezu, S Suzuki, A Nawa, M Kawai, T Nagasaka, F Kikkawa

**Affiliations:** 1 Department of Obstetrics and Gynecology, Nagoya University Graduate School of Medicine, 65 Tsuruma-cho, Showa-ku, Nagoya 466-8550, Japan; 2 Department of Obstetrics and Gynecology, Ehime University, Ehime, Japan; 3 Department of Obstetrics and Gynecology, Toyohashi Municipal Hospital, Aichi, Japan; 4 Nagoya University School of Health Science, Nagoya, Japan

**Keywords:** epithelial ovarian cancer, fertility-sparing surgery, overall survival, disease-free survival, grade

## Abstract

**Objectives:**

To compare the clinical outcome of patients with stage I epithelial ovarian cancer (EOC) who received with fertility-sparing surgery (FSS) with those who underwent radical surgery (RS).

**Methods:**

After a central pathological review and search of the medical records from multiple institutions, a total of 572 patients were retrospectively evaluated. All patients were divided into three groups: group A {FSS (*n*=74); age, ⩽40} groups B and C [RS; age, 40⩾{(B), *n*=52} 40<{(C), *n*=446}].

**Results:**

Five-year overall survival (OS) and disease-free survival (DFS) rates of patients in the groups were as follows: group A, 90.8% (OS)/87.9% (DFS); group B, 88.3% (OS)/84.4% (DFS); group C, 90.6% (OS)/85.3% (DFS), respectively (OS, *P*=0.802; DFS, *P*=0.765). Additionally, there was no significant difference in OS and DFS among the three groups stratified to stage IA or IC (OS (IA), *P*=0.387; DFS (IA), *P*=0.314; OS (IC), *P*=0.993; DFS (IC), *P*=0.990, respectively). Furthermore, patients with a grade 1–2 or 3 tumours in the FSS group did not have a poorer prognosis than those in the RS group.

**Conclusions:**

Stage I EOC patients treated with FSS showed an acceptable prognosis compared with those who underwent RS.

Epithelial ovarian carcinoma (EOC) is the leading cause of death from gynaecological malignancy (Greenlee *et al*, 2001). The conventional surgical modality for patients with early-stage EOC is based on hysterectomy and bilateral salpingo-oophorectomy with peritoneal sampling (peritoneal washing, omentectomy, multiple peritoneal biopsies, and the removal of peritoneal implants) regardless of lymph node resection. Several studies have estimated that 3–17% of all EOCs occur in women under 40 years of age (Smedley and Sikora, 1985; Swenerton *et al*, 1985; Plaxe *et al*, 1993; Rodriguez *et al*, 1994; Duska *et al*, 1999). In these young patients, the preservation of reproductive and/or female endocrine functions is an important issue. Commonly, fertility-sparing surgery (FSS) has been selected in young patients with borderline, germ cell, and stromal tumour, and several investigators have proposed the use of this surgical procedure for stage I/grade 1 invasive EOC. However, because of the risk of leaving an occult tumour in the remaining ovary and/or fear of impairing curability, most gynaecologists hesitate to adopt FSS in all stage I invasive EOCs. Indeed, in selecting FSS, the risk of recurrence and death may be increased. However, there is insufficient evidence to support this as most previous reports have not focused on the difference in survival between patients who received FSS and those who underwent radical surgery (RS).

Until now, we have encountered 74 EOC patients at stage I who desired to preserve their childbearing potential and underwent FSS. To explore indications for FSS in young patients with stage I EOC, the question of whether or not choosing FSS alters recurrence-free and overall survival (OS) was investigated comparing FSS-receiving patients with 498 patients who underwent radical surgery (RS).

## Patients and methods

### Patients and tumour status

A variety of malignant ovarian tumours have been accumulated by the Tokai Ovarian Tumour Study Group, consisting of Nagoya University and affiliated cooperating institutions, under the central pathological review system since 1986. Of these, 572 patients with stage I (FIGO, 1988) EOC, including 74 patients who had undergone FSS were registered between January 1986 and December 2007 were extracted. Borderline tumours were excluded in this study. Data were collected from the medical records and clinical follow-up visits. This study was approved by the ethics committee in Nagoya University.

Patients were excluded from this study when they showed insufficient clinical data or were lost to follow-up immediately after surgery. The histological cell types were assigned according to the criteria of the World Health Organisation (WHO). Histological slides were reviewed by one of the investigators under a central pathological review system with no knowledge of the patients’ clinical data.

Additionally, according to the patients with an FIGO stage IC classification, we defined two subtypes of pathological characteristics: IC(r) for patients with intraoperative capsule rupture and a negative cytology; IC (non-r) for those with IC excluding IC(r), including a tumour on the ovarian surface/preoperative capsule rupture, or with positive malignant cells in the positive peritoneal washing/ascites.

All EOC patients were divided into three groups: group A, patients who underwent FSS based on the criteria described below; groups B (under or equal to 40 years old) and C (over 41 years old), patients who underwent conventional RS, including, in principle, hysterectomy, and bilateral salpingo-oophorectomy with peritoneal staging (peritoneal washing, omentectomy, multiple peritoneal biopsies, and the removal of peritoneal implants) with retroperitoneal lymphadenectomy or sampling. If patients were at too advanced an age, with severe comorbidities, retroperitoneal lymphadenectomy was exceptionally omitted. If retroperitoneal lymphadenectomy was omitted, the absence of an enlarged lymph node >1 cm in diameter was confirmed by preoperative CT scan; if present, palpable nodes were appropriately sampled. In principle, group A patients were eligible if they fulfilled the following: (1) had histologically confirmed stage I EOC, (2) were ⩽40 years of age at the time of the initial diagnosis, (3) strongly desired to retain fertility, (4) in a preoperative counselling session, these women were informed of the possible risks and benefits of FSS, and signed a consent form, (5) conservation of the uterus and contralateral ovary and fallopian tube with at least a full peritoneal staging (cytology of peritoneal washing or ascites, careful palpation and inspection throughout the peritoneal cavity, and, if necessary, multiple peritoneal biopsies), and (6) systematic retroperitoneal lymphadenectomy, wedge resection of the remaining ovary, and omentectomy, were optional. If systematic retroperitoneal lymphadenectomy or sampling was omitted, the absence of enlarged lymph nodes of >1 cm in diameter was confirmed by a preoperative CT scan.

In all stage I patients, 446 were treated postoperatively with 3–6 cycles of adjuvant platinum-based chemotherapy. A total of 126 patients (22.0%) did not receive adjuvant platinum-based chemotherapy because of severe complications, the patients’ wishes, within the criterion of omission (stage IA/grades 1–2), and the decision of each institution. Details of the chemotherapy regimen in each period were described previously (Suzuki *et al*, 2008).

### Follow-up and analysis

At the end of treatment, all patients underwent a strict follow-up, consisting of clinical check-ups such as a pelvic examination, ultrasonographic scan, CA125 evaluation, and periodic CT scan. The OS was defined as the time between the date of surgery and that of the last follow-up or death because of EOC. Disease-free survival (DFS) was defined as the time interval between the date of surgery and that of recurrence or the last follow-up. The distributions of clinicopathologic events were evaluated using the *χ*^2^-tests. Univariate survival analysis was based on the Kaplan–Meier method. Comparison between the survival curves was conducted using the log-rank test. Multivariate analysis was analysed employing Cox's proportional hazard model. A *P*-value of <0.05 was considered significant.

## Results

### Patients’ characteristics

A total of 572 EOC patients at stage I were entered into this study. The median follow-up for surviving patients was 62.5 (4.8–256.5) months in group A, 66.7 (6.3–448.3) months in group B, and 62.8 (1.6–252.6) months in group C. Patient characteristics of groups A–C are summarised in Table 1. Among the 74 group A patients, 36 (48.6%) had IA disease, and 37 (50.0%) had IC disease. Regarding the IC substage distribution, there was no difference among the three groups (*P*=0.355). On the other hand, among the total of 498 patients who underwent RS (groups B–C), 155 patients (31.1%) had IA disease, and 337 (67.7%) had IC disease. With regard to histological types, the frequency of a clear-cell histology was higher in the RS groups (groups B–C) than in the FSS group (group A) (*P*=0.0002). In addition, the grade distribution or frequency of performing adjuvant platinum-based chemotherapy was similar between the FSS (group A) and RS (groups B–C) groups (grade: *P*=0.294, chemotherapy: *P*=0.266).

Nineteen patients underwent wedge resection of the contralateral ovary. Only one patient was up-staged to stage IB because of the postoperative pathological finding of an occult tumour. Five patients received cystectomy (two patients: laparoscopic surgery) as an initial surgery before secondary salpingo-oophorectomy. Retroperitoneal lymphadenectomy was performed in five patients. Although lymphadenectomy was less performed in the group A than groups B–C (*P*<0.0001), none were re-classified at a higher stage following the histological analysis of lymph nodes.

### Univariate survival analysis

**Figure 1:**
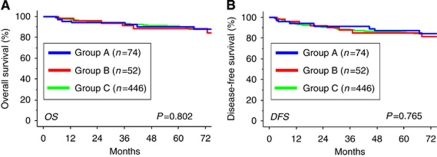
Kaplan–Meier estimated OS (**A**) or DFS (**B**) of all EOC patients at stage I. Survival curves were stratified by the surgical procedure and patients’ age. All EOC patients were divided into three groups: group A, patients who underwent FSS under 40 years old; groups B (under or equal to 40 years old) and C (over 41 years old), patients who underwent conventional RS, as described in ‘Materials and Methods section’ (group A (*n*=74), solid blue line; group B (*n*=52), solid red line; group C (*n*=446), solid green line).

The 5-year OS rates in the individual groups were as follows: group A, 90.8% group B, 88.3% and group C, 90.6%. There was no significant difference in OS among these groups (Figure 1A, *P*=0.802). In addition, the 5-year DFS rate of all group A patients was 87.9%, compared with 84.4% in group B, and 85.3% in group C. On Kaplan–Meier analysis, the difference in DFS among these groups was also nonsignificant (Figure 1B, *P*=0.765).

**Figure 2:**
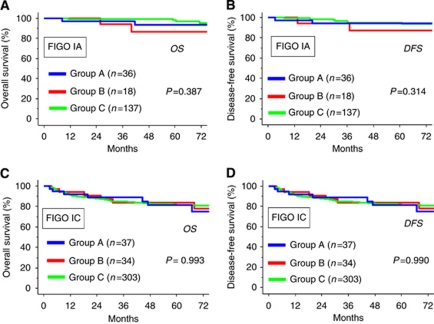
Kaplan–Meier estimated OS (**A** and **B**) or DFS (**C** and **D**) of patients who underwent FSS and RS stratified by FIGO stage (IA and IC). Grouping of patients is the same as shown in the legend of Figure 1.

Subsequently, we performed further survival analysis according to the stage I substage (IA and IC). Figure 2 shows the OS or DFS curves stratified by FIGO IA (OS: **A**, DFS: **B**) and FIGO IC (OS: **C**, DFS: **D**). Even when they were stratified by substage, there were no significant differences in survival among the three groups (OS (IA): *P*=0.387; DFS (IA): *P*=*0.*314; OS (IC): *P*=0.993; DFS (IC): *P*=0.990).

**Figure 3:**
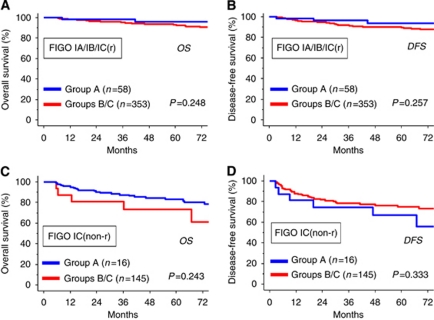
Kaplan–Meier estimated OS (**A** and **B**) or DFS (**C** and **D**) of patients who underwent FSS and RS stratified by FIGO IC substage (IC substage was defined as follows: IC(r): intraoperative capsule rupture, IC(non-r): IC excluding IC(r) such as preoperative capsule rupture or ascites/washing positive). Grouping of patients was as follows: FSS under or equal to 40 years old (group A: blue line) and RS in all ages (groups B/C: red line). (**A** and **B**): Kaplan–Meier curves of patients with IA/IB/IC(r), (**C** and **D**): Kaplan–Meier curves of patients with IC(non-r).

We further compared the survival between the FSS (group A) and RS (groups B–C) groups, when patients were stratified to either grouping of IA/IB/IC(r) or IC(non-r). As shown in Figures 3A and B, in patients with IA/IB/IC(r), there were also no differences in OS and DFS between the two groups. Similarly, even in patients with stage IC(non-r), no significant difference in both OS and DFS were identified (Figure 3C: OS: *P*=0.243, Figure 3D: DFS: *P*=0.333).

**Figure 4:**
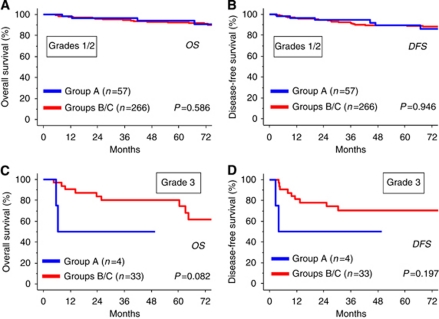
Kaplan–Meier estimated OS (**A** and **B**) or DFS (**C** and **D**) of patients who underwent FSS and RS stratified by the tumour grade. Grouping of patients is the same as shown in the legend of Figure 3.

In this study, we assessed the tumour grade in the stage I patients excluding clear-cell histology. We investigated whether there was any difference in survival between the FSS and RS groups, even when patients were stratified to each grade. Figures 4A and B shows that, for both OS and DFS, the prognosis of patients with grades 1–2 tumours in the FSS group was not significantly different from that in the RS group (grades 1–2: OS: *P*=0.586, DFS: *P*=0.946). In addition, there were four grade 3 EOC in the FSS group, and, of those, two (50%) showed recurrence and died within 12 months of the diagnosis, while those who underwent RS did not reach median DFS or OS within 6 years of follow-up. Although there was no difference in the survival of patients with grade 3 tumours between the two groups (grade 3: OS: *P*=0.082, DFS: *P*=0.197), the number of patients with grade 3 tumours was too small to arrive at a definite conclusion.

### Multivariate analysis

To eliminate selection bias from a number of clinicopathologic factors as thoroughly as possible, we finally performed multivariate OS/DFS analyses. The age, FIGO stage (IA *vs* IB–C), surgical procedure (FSS *vs* radical), histological type (clear-cell *vs* non-clear-cell), comprehensive full-staging surgery (absent *vs* present), preoperative CA125 value, postoperative adjuvant chemotherapy, and peritoneal cytology (positive *vs* negative) were entered into the uni- or multivariate OS/DFS analyses (Table 2). A clear-cell histology has been shown to be chemoresistant, and has a more malignant potential compared with other histological types. We considered this type of tumour as a separate entity for the indication of FSS. This was the reason for our classification of the histology as clear-cell *vs* non-clear-cell. Among the selected clinicopathologic factors for murtivariable analysis, the stage, chemotherapy regimen, and peritoneal cytology were significantly poorer prognostic factors for OS and DFS. However, the surgical procedure (FSS or RS) was not (Table 2: DFS, HR: 0.874, 95% CI: 0.361–2.115, *P*=0.765; OS, HR: 0.877, 95% CI: 0.335–2.297, *P*=0.789).

## Discussion

One potential risk of FSS is an increase in recurrence and death from disease. According to a number of previous studies reporting the outcome of patients who have undergone FSS, this procedure may be appropriate for early-stage EOC (Zanetta *et al*, 1997; Morice *et al*, 2001; Schilder *et al*, 2002; Kajiyama *et al*, 2008; Park *et al*, 2008; Satoh *et al*, 2010). However, the conclusion of each study generally focused, not on survival analysis, but on recurrence rates, and so the recommended criteria for FSS remain controversial. In addition, the results of most previous investigations were based on comparisons of clinical outcomes among patients who underwent FSS. In this context, it is an essential question whether or not the selection of FSS itself impairs the long-term outcome of early-stage EOC patients.

In this study, to assess the appropriateness of FSS, we compared the survival between patients who have undergone FSS and those who had received RS. Comparison between the FSS and RS groups revealed no difference in the OS and DFS between them, regardless of the stage I substage in the univariate analysis. Furthermore, the multivariate analysis, which we aimed to eliminate the selection bias, revealed that the surgical procedure was not an independent prognostic factor for stage I EOC. Consistent with our results, according to the large-scale retrospective analysis by Wright *et al* (2009), ovarian or uterine preservation had no effect on survival compared with a RS group. In this context, the current findings suggest that, among young patients with early-stage EOC, FSS appears to be safe and does not impair survival.

We previously showed that the survival of patients with stage IC associated with preoperative rupture or positive ascites was poorer than in those with stage IA, and that, on comparing patients with stage IA to those with IC(r), the difference in survival was not significant (Kajiyama *et al*, 2010). This suggests that patients with intraoperative capsule rupture are candidates for FSS; in contrast, preoperative rupture appeared to be one of the contraindicative factors for FSS.

Nevertheless, this study demonstrated that even in stage IC(non-r), there was no difference in OS and DFS between patients undergoing FSS and those receiving RS. Furthermore, Satoh *et al* (2010) independently revealed the possibility of FSS in IC(non-r) patients if they were followed by adjuvant chemotherapy. According to their results in the FSS cohort, 5-year OS and DFS of stage IC patients excluding clear-cell type and grade 3 tumours were 96.9% and 92.1%, respectively. We suggest that, under the condition of preoperative rupture/ascites/washing positive, occult chemoresistant metastasis is likely to have formed elsewhere in the peritoneal cavity, not in the remaining ovary or uterus alone. Thus, if occult metastases had already been present, RS, including hysterectomy and contralateral oophorectomy, may not have influenced the survival. However, the number of subjects in this study was too small to verify the actual appropriateness of FSS for patients with IC(non-r). Thus, at present, we mention this only as a possibility.

Our subsequent concern was the possibility of FSS in each tumour grade. Morice *et al* (2005) reported 10 cases of invasive recurrence in 33 patients with stage I EOC who had undergone FSS. Among those patients, there were six stage IA patients, one of which was at grade 1, four grade 2, and one showed a clear-cell histology. Thus, they recommend not performing FSS in patients beyond stage IA/G1. However, regardless of performing FSS, since grade 3 tumours were shown to lead to a poorer survival than those at grades 1/2 (Vergote *et al*, 2001), it is unknown whether the choice of FSS itself led to such a poorer prognosis. Moreover, previous reports indicated that grade assessment was subjective and there was considerable inter-observer variation in the diagnostic reproducibility of the grade in early-stage EOC (Baak *et al*, 1986; Bertelsen *et al*, 1993). Namely, once a different pathologist examines a slide, it is possible that a different grade is assigned. Therefore, it is recommended that all cases of early-stage EOC be reviewed by pathologist with expertise in gynaecologic pathology. In this context, one of the strengths of our study was that assessments of the grades and histological types were performed under the central pathological review system. Our current survival analyses were stratified based on both the surgical procedure and grade, patients in the FSS group did not necessarily have a poorer prognosis than those in the RS group, even in the patients with grade 3 tumours. Accordingly, in the above-mentioned report by Satoh *et al*, 2010, patients with grade 2 tumours were considered as candidates for FSS if they received adjuvant chemotherapy (Satoh *et al*, 2010). However, even in their analysis, they concluded that grade 3 tumour presence was a contraindication for FSS because of the result that three in six patients showed recurrence. Irrespective, in our current cohort, the number of patients with grade 3 tumours was too small to arrive at a definite conclusion at present. The lack of a significant difference in survival may be solely because of the low numbers. In any case, we cannot offer FSS to patients with a stage I/grade 3 tumour at present because both Satoh's and our data showed a 50% recurrence risk. Taken together, we should accumulate further cases to clarify treatment prospects.

In summary, our current investigation suggests that FSS is considered in young patients with stage IA/IC(r) and at least grades 1–2 EOC. However, this study was very preliminary and had several biases such as the small number of cases, the possibility of type II error, variable follow-up length, and different treatment protocols during the long-term study period. Furthermore, one of the major limitations of this study was that not all of the cases underwent systematic lymphadenectomy although there were no up-staged patients in this study. In several previous reports regarding FSS in EOC, lymphadenectomy was optional (Park *et al*, 2008; Satoh *et al*, 2010). However, we think that, in principle, comprehensive surgical staging is necessary for all patients who wish to receive FSS, because CT has a poor sensitivity for low-volume nodal disease. On this occasion, we merely provide a hypothesis that patients with stage I EOC who have undergone FSS may not show a poorer prognosis than those receiving RS. Concerning the patients’ specificity and ethical aspect, a randomised controlled study is unlikely. Thus, we hope that the hypothesis will be verified by accumulating further numbers of patients treated with FSS through a large-scale, clinical registry system developed in the near future.

## Figures and Tables

**Table 1:** Patients’ characteristics

		**FSS**	**Radical surgery**		
	**Total**	**Group A**	**Group B**	**Group C**	***P*-value**
Total	572	74	52	446	
					
*Age*					<0.0001
⩽40	126	74	52	0	
>40	446	0	0	446	
					
*FIGO stage*					0.0291
IA	191	36	18	137	
IB	7	1	0	6	
IC	374	37	34	303	
IC(r)^*1^	213	21	21	171	
IC(non-r)^*2^	161	16	13	132	
					
*Histological type*					0.0002^#1^
Serous	64	4	4	56	
Mucinous	150	43	18	89	
Clear-cell	212	13	17	182	
Endometrioid	128	14	11	103	
Others^*3^	18	0	2	16	
					
*Grade*					0.294^#2^
G1/G2	323	57	33	233	
G3	37	4	2	31	
NC^*4^	212	13	17	182	
					
*Comprehensive surgical staging surgery*					NA
Yes	321	NC	24	297	
No	177	NC	28	149	
FSS	74	74	NC	NC	
					
*Preoperative CA125 value*					0.0003
⩽35 IU ml^–1^	204	29	14	161	
>35 IU ml^–1^	332	33	37	262	
Unknown	36	12	1	23	
					
*Peritoneal cytology*					0.145
Negative	407	58	35	314	
Positive	165	16	17	132	
					
*Platinum-based chemotherapy*					0.266^#3^
Taxane plus platinum	241	22	16	203	
Conventional platinum-based	205	32	30	143	
None	126	20	6	100	

Abbreviations: FIGO=International Federation of Gynecology and Obstetrics; FSS=fertility-sparing surgery; NC=not classified; NA=not analysed; ^*^1=intraoperative capsule rupture; ^*^2=washing/ascites positive or preoperative capsule rupture; ^*^3=mixed epithelial tumour and undifferentiated carcinoma; ^*^4=grade of clear-cell pathology was not classified. *P*-values: comparison of groups A with groups B–C; #1=Clear-cell *vs* non clear-cell, #2=G1/G2 *vs* G3; #3=platinum-based chemotherapy, present *vs* absent.

**Table 2:** Uni- and multivariate analyses of clinicopathologic parameters in relation to OS and DFS of patients

		**OS**				**DFS**			
		**Univariate analysis**		**Multivariate analysis**		**Univariate analysis**		**Multivariate analysis**	
	**No**	**5-Year OS (%)**	***P*-value**	**Hazard ratio (95% CI)**	***P*-value**	**5-Year DFS (%)**	***P*-value**	**Hazard ratio (95% CI)**	***P*-value**
Total	572								
									
*Age*									
⩽40	126	89.8	0.956	1		86.5	0.969	1	
>40	446	90.6		0.882 (0.430–1.811)	0.7322	85.3		0.864 (0.440–1.697)	0.671
									
*FIGO* *stage*									
IA	191	96.1	0.0007	1		93.9	<0.0001	1	
IB/C	381	87.5		2.776 (1.314–5.866)	0.0074	81.3		2.898(1.472–5.703)	0.0021
									
*Surgery*									
Radical	498	90.4	0.663	1		85.2	0.592	1	
FSS	74	90.8		0.877 (0.335–2.297)	0.789	87.9		0.874 (0.361–2.115)	0.7652
									
*Comprehensive surgical staging surgery*									
Yes	321	90.8	0.7387	NA		86.0	0.6861	NA	
No	177	89.5				83.7			
FSS	74	90.8				87.9			
									
*Histology*									
Clear-cell	212	88.3	0.966	1		87.4	0.281	1	
Non-clear-cell	360	91.7		1.065 (0.646–1.757)	0.8045	82.5		0.843 (0.545–1.306)	0.4449
									
*Cytology*									
Negative	407	92.7	0.0008	1		88.8	0.0001	1	
Positive	165	84.2		1.760 (1.061–2.918)	0.0284	77.2		1.815 (1.156–2.850)	0.0096
									
*CA125 value* (*U ml*^*–1*^*)*									
⩽35 or unknown	240	94.1	0.0158	NA		91.1	0.003	NA	
>35	332	87.8				81.6			
									
*Platinum-based chemotherapy*									
Taxane plus platinum	241	92.8	0.0269	1		87.0	0.0415	1	
Conventional platinum-based	205	84.5		2.036 (1.027–2.729)	0.0194	80.7		1.674 (1.027–2.729)	0.0387
None	126	96.4		2.152 (0.861–5.381)	0.1011	90.3		1.924 (0.891–4.155)	0.0957

Abbreviations: CI, confidence interval; DFS, disease-free survival; FIGO=International Federation of Gynecology and Obstetrics; FSS, fertility-sparing surgery; NA, not analysed; OS, overall survival.
